# Long-term prediction of $^{137}$Cs in Lake Onuma on Mt. Akagi after the Fukushima accident using fractional diffusion model

**DOI:** 10.1038/s41598-021-99667-1

**Published:** 2021-10-13

**Authors:** Eiichi Suetomi, Yuko Hatano, Masakiyo Fujita, Yukiko Okada, Kyuma Suzuki, Shun Watanabe

**Affiliations:** 1 Faculty of Engineering, Information and Systems, University of Tsukuba, 1-1-1 Tennodai, Tsukuba, Ibaraki 305-8573 Japan; 2 Faculty of Engineering, Information and Systems; Center for Research in Isotopes and Environmental Dynamics, University of Tsukuba, 1-1-1 Tennodai, Tsukuba, Ibaraki 305-8573 Japan; 3 Graduate School of Science and Technology, University of Tsukuba, 1-1-1 Tennodai, Tsukuba, Ibaraki 305-8573 Japan; 4 Atomic Energy Research Laboratory, Tokyo City University, Ozenji 971, Asao-ku, Kawasaki, Kanagawa 215-0013 Japan; 5 Gunma Prefectural Fisheries Experiment Station, 13, Shikishima, Maebashi, Gunma 371-0036 Japan

**Keywords:** Environmental sciences, Limnology, Applied mathematics

## Abstract

The Fukushima Daiichi Nuclear Power Plant accident also contaminates lakes in Japan. Especially in closed lakes, there is a problem of prolonged low-level $^{137}$Cs contamination because the activity concentration of $^{137}$Cs declines sharply immediately after the accident, but then begins to decrease slowly. In this paper, we derived a long-term prediction formula based on the fractional diffusion model (FDM) for the temporal variation in $^{137}$Cs activity concentrations of the water in Lake Onuma on Mt. Akagi, one of the closed lakes, and of pond smelt (Hypomesus nipponensis), a typical fish species inhabiting in the lake. The formula reproduced well the measured $^{137}$Cs activity concentration of the lake water and pond smelt for 5.4 years after the accident. Next, we performed long-term prediction for 10,000 days using this formula and compared it with the prediction results of the two-component decay function model (TDM), which is the most common model. The results suggest that the FDM prediction will lead to a longer period of contamination with low-level $^{137}$Cs than the TDM prediction.

## Introduction

During the Fukushima Daiichi Nuclear Power Plant (FDNPP) accident, aquatic ecosystems are contaminated with radioactive $^{137}$Cs^1–6^. At present, the activity concentration of $^{137}$Cs in river water is quite low^7–9^. However, in closed lakes, there is a problem of long-tailed temporal change in low-level radioactive contamination because the activity concentration declines steeply immediately after the accident, but then turns to a gradual decrease^6,10^. Such a problem is also observed in closed lakes and small lakes after the Chernobyl accident^11,12^. Lake Onuma on Mt. Akagi in Gunma Prefecture located about 190 km from the FDNPP is a semi-closed system with limited amount of inflow and runoff water and an average water residence time of 2.3 years^10^. In August 2011, $^{134}$Cs plus $^{137}$Cs activity concentration of pond smelt (Hypomesus nipponensis), a typical fish species inhabiting in Lake Onuma exceeded 500 Bq ${\text {kg}}^{-1}$ the standard limits of radiocesium of foods in Japan at that time. As a result, the future of radioactive contamination in closed lakes became a matter of public concern. Therefore, the $^{137}$Cs activity concentrations of the lake water and pond smelt in Lake Onuma were measured for a long period of 5.4 years after the accident^10^.

The main processes governing $^{137}$Cs activity concentration after the accident can be categorized into an initial phase and mid-, to long-term phases, corresponding to the post-accident phase. The most important processes in the initial phase are the atmospheric fallout of $^{137}$Cs on the water surface, subsequent sorption and fixation by sediments, and their settling on the bottom^13–15^. Besides pure fitting, a parameter for a given lake and radionuclide in the diffusion model can be estimated using characteristics with clear meaning $^{137}$Cs deposition, effective diffusion coefficient, and distribution coefficient^11^, and thus predictions of $^{137}$Cs long-term dynamics in a lake can be provided immediately after the accident using previously measured values of the characteristics, literature data or expert judgments. Studies in the mid-, to long-term phases after the accident include models using sum of exponential functions^13,16,17^ and process-level diffusion models^11,18,19^. The two-component decay function model (TDM)^16,17^, which is the sum of two exponential functions, was used to fit the measured $^{137}$Cs activity concentration and showed good agreement with the measured values^6,10,12^. On the other hand, by using the diffusion models, Bulgakov et al.^11^ and Konoplev et al.^18,19^ succeeded in showing the temporal trend of radiocesium in the mid- and long-term phases after the accidents at Chernobyl and Fukushima.

Various factors are considered to be responsible for the temporal variation in $^{137}$Cs activity concentration of lake water, including sedimentation on the lake sediment due to adsorption on inorganic particles, elution from the lake sediment^20,21^, circulation in the lake due to convection^22–25^, turbulent mixing^26^, absorption by plankton and other organisms, and many other factors are possible. A model that describes such complex diffusion is the fractional diffusion model (FDM)^27,28^, which includes a fractional order differentiation^29^ with respect to time. The FDM has been used in the study of actual mass transfer to explain phenomena that are different from classical diffusion, especially those that diffuse slower than the classical diffusion. Some of the earliest engineering applications of the FDM include studies of concentration of an electroactive species at the surface of an electrode^30^ and transient photocurrent in inorganic and organic amorphous materials^31^. Other examples include applications to fluids in porous media^32^ and sorption and convection of ions in heterogeneous media^33,34^ and studies on turbulence^35–37^. Based on these backgrounds, we will try to reproduce the temporal variation in $^{137}$Cs activity concentration of the water in Lake Onuma using the FDM.

The actual measurement period of the water in Lake Onuma is the one of the longest for a closed lake in Japan, 5.4 years after the FDNPP accident. However, there are less measured values of $^{137}$Cs after that period. Since there are less measured values of $^{137}$Cs activity concentration of the water and pond smelt in Lake Onuma since 5.4 years after the accident and not publicly available, the long-term temporal changes in $^{137}$Cs activity concentration of the lake water and pond smelt over 10,000 days since the accident have not been clarified.

The main goals of the present study are to (1) derive a long-term prediction formula for the temporal variation in $^{137}$Cs activity concentration of the water and pond smelt in Lake Onuma based on the FDM, (2) conduct long-term prediction of $^{137}$Cs activity concentration of the water and pond smelt in Lake Onuma for 10,000 days using this prediction formula, (3) clarify the difference between the predictions of the FDM and of the TDM. Such a prediction formula will enable long-term prediction of radioactive contamination in closed lakes and provide long-term prospects to the residents living in the vicinity, thereby relieving their psychological anxiety and preventing the spread of rumors.

## Mathematical modeling

Roche et al.^38^ used the continuous time random walk (CTRW)^39^ to analyze the mass transport within the sediments of Lake DePue, a backwater lake of the Illinois River. The results showed that the CTRW could reproduce the concentration in the sediments, especially from the sediment interior at the lake bottom to the water interface. On the other hand, the present study examined the applicability of the CTRW up to the upper water above the sediment. Based on the study of Metzler et al.^40^, we generalized diffusion of $^{137}$Cs from the CTRW to the fractional diffusion model (FDM), and derived the fractional diffusion equation (FDE). The derivation of the FDE is described below.

In the CTRW, the jump length of a particle ($^{137}$Cs) and the waiting time between jumps are represented by a probability density function (PDF) $\psi (x, t)$. We consider the jump length PDF $\lambda (x)$ and the waiting time PDF *w*(*t*) are independent variables, and one finds the separation of variable $\psi (x, t) = \lambda (x) w(t)$.We describe the CTRW model using $\psi (x, t)$ by the following master equation^39^:

1 $$\begin{aligned} \eta (x, t) = \int dx' \int \eta (x', t') \psi (x-x', t-t') dt' + \delta (t) \delta (x). \end{aligned}$$

where $\eta (x, t)$ is the PDF when the particle arrives at time *t*, position *x*, and the second term on the right-hand side corresponds to the initial condition of the random walk.

We Fourier transform Eq. (1) from position *x* to *k* and obtain ${\tilde{\eta }}(k , t)$. We Laplace transform ${\tilde{\eta }}(k , t)$ from time *t* to *s* and obtain $\hat{ {\tilde{\eta }} }(k , s)$. In this study, the Fourier transform and the inverse Fourier transform are defined, respectively, as

2 $$\begin{aligned} {\fancyscript {F}}[f(x)] = {\tilde{f}}(k) = \int _{-\infty }^{\infty } f(x) e^{2 \pi i kx} dx, \end{aligned}$$

and

3 $$\begin{aligned} {\fancyscript {F}}^{-1} [{\tilde{f}}(k)] = f(x) = \int _{-\infty }^{\infty } {\tilde{f}}(k) e^{-2 \pi i kx} dk. \end{aligned}$$

The Fourier-Laplace transform of Eq. (1) leads to the solution

4 $$\begin{aligned} \hat{ {\tilde{\eta }} }(k, s) = \frac{ 1 }{ 1 - \hat{ {\tilde{\psi }} }(k, s) }. \end{aligned}$$

Now applying the method of separation of variables and Fourier-Laplace representation, the PDF $\psi (x, t)$ in Eq. (1) can be written as follows:

5 $$\begin{aligned} \hat{ {\tilde{\psi }} }(k, s) = {\tilde{\lambda }}(k) {\hat{w}}(s), \end{aligned}$$

where ${\tilde{\lambda }}(k)$ and ${\hat{w}}(s)$ are the jump length PDF in the Fourier space and waiting time PDF in the Laplace space, respectively.

Let $\Phi (t)$ be the survival probability that the waiting time on a site exceed *t*:

6 $$\begin{aligned} \Phi (t) = 1 - \int _{0}^{t} w(t') dt'. \end{aligned}$$

The Laplace transform of $\Phi (t)$ corresponds to

7 $$\begin{aligned} {\hat{\Phi }} (s) = \frac{ 1 - {\hat{w}} (s) }{ s }. \end{aligned}$$

Equation (1) leads to the PDF of *C*(*x*, *t*) for $^{137}$Cs at *x* at time *t* is given by

8 $$\begin{aligned} C(x, t) = \int dx' \int \eta (x', t') \Phi (x-x', t-t') dt'. \end{aligned}$$

Combining Eqs. (4), (5) and (7), we get the Fourier-Laplace transform of the PDF *C*(*x*, *t*):

9 $$\begin{aligned} \hat{ {\tilde{C}} }(k, s) = \hat{ {\tilde{\eta }} }(k, s) {\hat{\Phi }}(s) = \frac{ {\hat{\Phi }}(s) }{ 1 - \hat{ {\tilde{\psi }} }(k, s) } = \frac{ 1 }{ 1 - {\tilde{\lambda }}(k) {\hat{w}}(s) } \frac{ 1 - {\hat{w}}(s) }{ s }. \end{aligned}$$

Let us consider a situation in which $\lambda (x)$ behaves as a Gaussian jump length PDF:

10 $$\begin{aligned} \lambda (x) = \frac{1}{\sqrt{4 \pi \sigma ^2}} \exp \left[ -x^2 / ( 4 \sigma ^2 ) \right] , \end{aligned}$$

where $\sigma ^2$ is given by

11 $$\begin{aligned} \sigma ^2 = \frac{1}{2} \int _{-\infty }^{\infty } x^2 \lambda (x) dx. \end{aligned}$$

We Fourier transform $\lambda (x)$ and expanded to the order of $k^2$, Eq. (10) can be approximated by the following formula:

12 $$\begin{aligned} {\tilde{\lambda }}(k) \sim 1 - \sigma ^2 k^2 . \end{aligned}$$

Suzuki et al.^10^ reported the decay processes of $^{137}$Cs activity concentration of the lake water in Lake Onuma were well suited by not the single-component decay function model (SDM) but the two-component decay function model (TDM) between 234–1981 days from March, 15, 2011. This implies that there is a long-tailed (heavy-tailed) distribution with respect to time *t*. Accordingly, we assume that the PDF of waiting time *w*(*t*) behaves asymptotically as a power law, and *w*(*t*) is described as follows^31,41,42^:

13 $$\begin{aligned} w(t) \sim \frac{ \alpha }{ \Gamma ( 1 - \alpha ) }\frac{ \tau ^{\alpha } }{ t^{1 + \alpha } }, \; \; \; {\text {for}} \; \; \; 0< \alpha < 1, \end{aligned}$$

where

14 $$\begin{aligned} \tau = \int _{0}^{\infty } t \, w(t) dt. \end{aligned}$$

The Laplace transform of Eq. (13) is given by the following expression for a small *s* (corresponding to a large time *t*)^41^:

15 $$\begin{aligned} {\hat{w}}(s) = 1 - \tau ^{\alpha } s^{\alpha }. \end{aligned}$$

Substituting Eqs. (12) and (15) into the Eq. (9) gives:

16 $$\begin{aligned} \hat{ {\tilde{C}} }(k, s) = \frac{ 1 }{ \left( \sigma k \right) ^2 + \left( \tau s \right) ^{\alpha } - \sigma ^2 \tau ^{\alpha } k^2 s^{\alpha } } \frac{ \left( \tau s \right) ^{\alpha } }{ s }. \end{aligned}$$

We can neglect the mixed term $\sigma ^2 \tau ^{\alpha } k^2 s^{\alpha }$ of the higher order^41^ and obtain

17 $$\begin{aligned} \hat{ {\tilde{C}} }(k, s) = \frac{ 1 }{ s } \frac{ 1 }{ 1 + K_{\alpha } s^{-\alpha } k^2 }, \end{aligned}$$

where

18 $$\begin{aligned} K_{\alpha } = \frac{\sigma ^2}{\tau ^{\alpha }}. \end{aligned}$$

In order to derive FDE from Eq. (17), we employ the fractional derivative of an arbitrary order $\alpha$ defined by so-called Riemann–Liouville fractional integral as follows:

19 $$\begin{aligned} {_{0} D_{t}^{\alpha } } \, f(t) = \frac{1}{ \Gamma ( n - \alpha ) } \frac{d^{n}}{dt^{n}} \int _{0}^{t} \frac{ f(t') }{ (t - t')^{1 - n + \alpha } } dt', \end{aligned}$$

where *n* is positive integer and $n - 1 \le \alpha < n$. Employing the integral rule for the Laplace transform of fractional derivative^29^

20 $$\begin{aligned} {\mathcal {L}} \left[ { _{0} D_{t}^{q} } \, f(t) \right] = {\mathcal {L}} \left[ \frac{d^{q}}{dt^{q}} f(t) \right] = \frac{1}{ \Gamma (-q ) } {\mathcal {L}} \left[ t^{-1-q} \right] {\mathcal {L}} [f(t)] = s^q {\hat{f}} (s), \; \; \; q < 0, \end{aligned}$$

we inverse Laplace transform from *s* to *t* in Eq. (17):

21 $$\begin{aligned} {\tilde{C}} (k, t) + K_{\alpha } \, { _{0} D_{t}^{-\alpha } } \, k^2 {\tilde{C}} (k, t) = 1, \end{aligned}$$

and inverse Fourier transform from *k* to *x* in Eq. (21) leads to the following expression for *C*(*x*, *t*):

22 $$\begin{aligned} C(x, t) - \delta (x) = K_{\alpha } \, { _{0} D_{t}^{-\alpha } } \, \frac{ \partial ^2}{ \partial x^2} C(x, t). \end{aligned}$$

Let us apply the differential operator $\partial / \partial t$ to Eq. (22), we finally obtain the FDE:

23 $$\begin{aligned} \frac{\partial }{\partial t} C(x, t) = K_{\alpha } \, { _{0} D_{t}^{1-\alpha } } \, \frac{ \partial ^2}{ \partial x^2} C(x, t) , \; \; \; 0< \alpha < 1. \end{aligned}$$

Our next step is to find the solution of Eq. (23). We look for the solution in the form:

24 $$\begin{aligned} C(x, t) = \sum _{n=0}^{\infty } a_{n} C_{n} (x, t), 
\end{aligned}$$

with expansion coefficients $a_{n}$ dep ending on the initial conditions and use the separation of variables ansatz:

25 $$\begin{aligned} C_{n} (x, t) = X_{n} (x) \cdot T_{n} (t). \end{aligned}$$

Substituting Eq. (25) into Eq. (23), one obtains

26 $$\begin{aligned} X_{n} (x) \, {\dot{T}}_{n} (t) = K_{\alpha } \, { _{0} D_{t}^{1-\alpha } } \, T_{n} (t) \cdot {\ddot{X}}_{n} (x) , \end{aligned}$$

where Eq. (26) can be rewritten as follows:

27 $$\begin{aligned} \frac{1}{ K_{\alpha } } \frac{ {\dot{T}}_{n} (t) }{ { _{0} D_{t}^{1-\alpha } } \, T_{n} (t) } = \frac{ {\ddot{X}}_{n} (x) }{ X_{n} (x) } = -\mu _{n}^{2}. \end{aligned}$$

From Eq. (27), we get the following decoupled set for the equation for the spatial and the temporal eigenfunctions using the constant value $-\mu _{n}^{2} \; (\mu _{n} > 0)$:

28 $$\begin{aligned} \frac{ d^2}{ d x^2} X_{n} (x) = -\mu _{n}^{2} X_{n} (x), \end{aligned}$$

and

29 $$\begin{aligned} \frac{d}{ d t} T_{n} (t) = -\mu _{n}^{2} K_{\alpha } \, { _{0} D_{t}^{1-\alpha } } \, T_{n} (t). \end{aligned}$$

The general solution of Eq. (28) is given as follows:

30 $$\begin{aligned} X_{n} (x) = {A_{c}}_{n} \cos ( \mu _{n} x ) + {A_{s}}_{n} \sin ( \mu _{n} x ), \end{aligned}$$

where prefactors ${A_{c}}_{n}$ and ${A_{s}}_{n}$ should be determined by the boundary conditions.

The solution to Eq. (29) is given through the Mittag-Leffler function $E_{\alpha } (z)$^43,44^ :

31 $$\begin{aligned} E_{\alpha } (z) = \sum _{k=0}^{\infty } \frac{ z^k }{ \Gamma ( \alpha k + 1) }, \end{aligned}$$

and written as follows:

32 $$\begin{aligned} T_n (t) = T_n (0) \, E_{\alpha } \left( - \mu _{n}^{2} \sigma ^{2} \left( \frac{ t }{ \tau } \right) ^{\alpha } \right) = T_n (0) \, E_{\alpha } \left( - \xi _{n} \left( \frac{ t }{ \tau } \right) ^{\alpha } \right) , \end{aligned}$$

where

33 $$\begin{aligned} \xi _{n} = \mu _{n}^{2} \sigma ^{2}. \end{aligned}$$

The full solution of the FDE (Eq. (23)) is given by the sum over all eigenfunctions, i.e., by

34 $$\begin{aligned} C(x, t) = \sum _{n=1}^{\infty } \left\{ {A_{c}}_{n} \cos ( \mu _{n} x ) + {A_{s}}_{n} \sin ( \mu _{n} x ) \right\} \, T_n (0) \, E_{\alpha } \left( - \xi _{n} \left( \frac{ t }{ \tau } \right) ^{\alpha } \right) . \end{aligned}$$

Since our purpose is to make long-term predictions, the fundamental mode $(n=1)$ in Eq. (34) becomes dominant for a sufficiently large time *t*. Therefore, *C*(*x*, *t*) can be approximately written as follows:

35 $$\begin{aligned} C(x, t) \approx \left\{ {A_{c}}_{1} \cos ( \mu _{1} x ) + {A_{s}}_{1} \sin ( \mu _{1} x ) \right\} \, T_1 (0) \, E_{\alpha } \left( - \xi \left( \frac{ t }{ \tau } \right) ^{\alpha } \right) . \end{aligned}$$

where $\xi = \xi _{1}$. Now, let us consider averaging Eq. (35) over space (distance *x*) to make time-dependent function, therefore we get an equation based on the FDM as follows:

36 $$\begin{aligned} C_{FDM} (t)= & {} \frac{T_1 (0)}{L} \int _{0}^{L} \left\{ {A_{c}}_{1} \cos ( \mu _{1} x ) + {A_{s}}_{1} \sin ( \mu _{1} x ) \right\} dx \, E_{\alpha } \left( - \xi \left( \frac{ t }{ \tau } \right) ^{\alpha } \right) \, \exp ( - \lambda _{d} \, t ) \nonumber \\= & {} A \, E_{\alpha } \left( - \xi \left( \frac{ t }{ \tau } \right) ^{\alpha } \right) \, \exp ( - \lambda _{d} \, t ), \end{aligned}$$

where $\lambda _{d}$ is the radioactive decay constant per day of $^{137}$Cs and

37 $$\begin{aligned} A = \frac{T_1 (0)}{L} \int _{0}^{L} \left\{ {A_{c}}_{1} \cos ( \mu _{1} x ) + {A_{s}}_{1} \sin ( \mu _{1} x ) \right\} dx. \end{aligned}$$

When we calculated using Eq. (36), it becomes numerically unstable as the value of the series expansion term *k* in Eq. (31) increased. In order to avoid this phenomenon, the formula proposed by Gorenflo et al.^45^ may be used for the calculation of the Mittag-Leffler function. That is, if $0<\alpha <1$ and $- t^{\alpha } < 0$, then $E_{\alpha } (-t^{\alpha })$ can be rewritten as:

38 $$\begin{aligned} E_{\alpha } (-t^{\alpha }) = \int _{0}^{\infty } \frac{1}{ \pi \alpha } e^{ - r^{1/\alpha } } \frac{ t^{\alpha } \sin ( \pi \alpha ) }{ r^{2} + 2r t^{\alpha } \cos ( \pi \alpha ) + t^{2 \alpha } } dr. \end{aligned}$$

To calculate the Eq. (36), replace *t* in Eq. (38) with $\xi ^{1/\alpha } (t / \tau )$. Equation (36) is thus

39 $$\begin{aligned} C_{FDM} (t) = A \int _{0}^{\infty } \frac{1}{ \pi \alpha } e^{ - r^{1/\alpha } } \frac{ \xi ( t/\tau )^{\alpha } \sin ( \pi \alpha ) }{ r^{2} + 2r \xi ( t/\tau )^{\alpha } \cos ( \pi \alpha ) + {\xi }^{2} ( t/\tau )^{2 \alpha } } dr \, \exp ( - \lambda _{d} \, t ), \end{aligned}$$

We calculated the integral in Eq. (39) by using SciPy^46^ which is an open-source scientific computing library for the Python programming language.

The physical meaning of the four parameters is explained below. The parameter *A* is the initial average activity concentration of $^{137}$Cs (Bq $\text {m}^{-3}$) in the whole lake. The parameter *A* is averaged with respect to the water depth, as defined in Eq. (37). The parameter $\tau$ represents the speed of diffusion: a large value of $\tau$ represents slow diffusion, while a small value of $\tau$ represents fast diffusion. Specifically, it is expressed in Eq. (14) as the average value of the waiting time. The parameter $\xi$ is given by Eq. (33). The parameter $\mu$ on the right hand side of Eq. (33) is related to the water depth of the lake. On the other hand, $\sigma ^{2}$ on the right hand side of Eq. (33) gives the variance of the jump lengths of a random walker, i.e. the variance related to how far it travels per unit time when it diffuses from the bottom of the lake (Eq. (11)). The parameter $\xi$ is proportional to $\sigma ^{2}$ and thus represents how fast individual $^{137}$Cs moves through the lake water. The vorticity diffusion coefficient ($K_{\alpha }$) in the lake water is given in the literature from observations in lakes all over the world and is defined by Eq. (18). The parameter $\alpha$ is the most important parameter and its value determines the overall behaviour of the diffusion. The value $\alpha$ indicates the extent to which the diffusion differs from the classical behaviour. This can only be determined by fitting.

## Results and discussion

In the following discussion, $^{137}$Cs activity concentration refers to the total $^{137}$Cs activity concentration (sum of dissolved and particulate $^{137}$Cs activity concentrations). The total $^{137}$Cs activity concentration is the average concentration of measurements at 0 m, 8 m and 15 m in the water column at the center of the lake^10^. Although total $^{137}$Cs activity concentration is very much variable and dependent on suspended matter concentration in the lake water, our main concern is the radiation safety of the lake water. That is why we use the total activity concentration in the present study.

The prediction formulae for $^{137}$Cs activity concentration used for comparison with the measured value are the fractional diffusion model (FDM) given by Eq. (39) and the two-component decay function model^10^ (TDM) written as follows:

40 $$\begin{aligned} C_{TDM} (t) = Q_{1} \exp (- k_{1} t ) + Q_{2} \exp (- k_{2} t ), \end{aligned}$$

where $Q_{1}$, $k_{1}$, $Q_{2}$ and $k_{2}$ are fitting parameters.

In order to determine the fitting parameters of Eqs. (39) and (40), the prediction formulae for $^{137}$Cs activity concentration of the water in Lake Onuma were fitted to the measured values. For all prediction formulae, we used the Levenberg–Marquardt algorithm to find the fitting parameters so that the squared relative error $\epsilon ^{2}$, defined by the following equation is minimized.

41 $$\begin{aligned} \epsilon ^{2} = \sum _{i=1}^{imax} \left( \frac{ C_{ m_{i} } - C( t_{i}) }{ C_{ m_{i} } } \right) ^{2}. \end{aligned}$$

where $C_{ m_{i} }$ is the *i*th measurement value and $C( t_{i})$ is the fitting value at time $t_{i}$.

**Figure 1:**
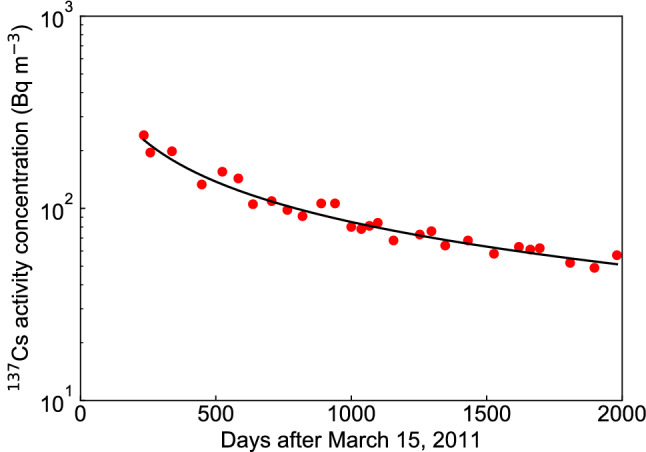
Change in the $^{137}$Cs activity concentration of water in Lake Onuma using the fractional diffusion model (FDM). The solid red circles indicate the measured $^{137}$Cs activity concentration found in the supplementary data^10^.

The FDM reproduced well the temporal variation in the measured $^{137}$Cs activity concentration (Bq ${\text {m}}^{-3}$) of the water in Lake Onuma collected in 2011–2016 (see Fig. 1). The solid line (FDM) in Fig. 1 is the result of fitting Eq. (39) with the unknown parameters (*A*, $\alpha$, $\xi$ and $\tau$) to the measured values. The values of the fitting parameters were $A = 1256$, $\alpha = 0.62$, $\xi = 5.00$ and $\tau = 693$. The solid red circles indicate the measured $^{137}$Cs activity concentrations listed in the supplementary data^10^. The value of $\epsilon ^{2}$ for the FDM was 0.207, and the $\epsilon ^{2}$ of the TDM was 0.208 (Table 1). In order to reproduce the decay process of $^{137}$Cs activity concentration, the TDM required two exponential functions, while the FDM required only the Mittag-Leffler function. Since the fitting formula based on the FDM reproduced well the temporal change in the measured $^{137}$Cs activity concentration in Lake Onuma, Eq. (39) was used as a prediction formula for the long-term prediction of $^{137}$Cs activity concentration.

**Table 1:** Values of the squared relative errors obtained as a result of fitting with each prediction models.

Model	$\epsilon ^{2}$
Fractional diffusion model (FDM)	0.207
Two-component decay function model (TDM)	0.208

On the other hand, a prediction formula based on the Bulgakov model^11^ is as follows:

42 $$\begin{aligned} C_{B} (t) = A_{B} \, t^{ -1/2 } \, \exp ( - \lambda _{d} t ), \end{aligned}$$

where $A_{B}$ is a fitting parameter, $\lambda _{d}$ is the radioactive $^{137}$Cs decay constant per day. Furthermore, a comparison between the FDM (Eq. (39)) and the Bulgakov model (Eq. (42)) showed that the FDM was a generalization of the Bulgakov model. Bulgakov et al.^11^ derived the $^{137}$Cs activity concentration in the lake water as follows:

43 $$\begin{aligned} C_{B} (t) = \frac{ \sigma _{d} }{ h_{w} } \exp ( z^{2} ) \, \text {erfc} (z) \, \exp ( - \lambda _{d} t ), \end{aligned}$$

where $C_{B} (t)$ is the activity concentration of $^{137}$Cs in the lake water at time *t*, $\sigma _{d}$ is the radionuclide deposition density, and $h_{w}$ is the average depth of the water column. The variable *z* in Eq. (43) is expressed as follows:

44 $$\begin{aligned} z = \frac{ K_{d} \sqrt{ D_{E} } }{ h_{w} } t^{1/2}, \end{aligned}$$

here, $D_{E}$ is the effective diffusion coefficient in sediments, $K_{d}$ is the dimensionless distribution coefficient. Bulgakov et al.^11^ derived Eq. (42) by approximating ${\text {erfc}} (z)$ in Eq. (43) as follows:

45 $$\begin{aligned} {\text {erfc}} (z) \approx \frac{ \exp ( -z^{2} ) }{ \sqrt{ \pi } } \, \frac{ 1 }{ z }. \end{aligned}$$

The following relation holds between the Mittag-Leffler function and the function $\exp ( z^{2} ) \, {\text {erfc}} (z)$ in Eq. (43)^47^:

46 $$\begin{aligned} E_{1/2} (-z) = \exp ( z^{2} ) \, {\text {erfc}} (z). \end{aligned}$$

Substituting $E_{1/2} (-z)$ in Eq. (46) into Eq. (43), we obtain the following equation:

47 $$\begin{aligned} C_{B} (t) = \frac{ \sigma _{d} }{ h_{w} } \times E_{1/2} \left( - \frac{ K_{d} \sqrt{ D_{E} } }{ h_{w} } t^{1/2} \right) \, \exp ( - \lambda _{d} t ). \end{aligned}$$

Thus, the FDM is a generalization of the Bulgakov model^11^. In other words, $\alpha$ in the Mittag-Leffler function in the Bulgakov model is 1/2, whereas the value of $\alpha$ in the FDM (Eqs. (36) and (39)) is in the range of $0< \alpha < 1$. For large time *t* the following relation holds^48^:

48 $$\begin{aligned} E_{\alpha } (-t^{\alpha }) \propto t^{- \alpha }. \end{aligned}$$

Therefore, Eq. (42) is reasonable for sufficiently large *t*, but when *t* becomes small, the approximation does not hold. Furthermore, in the FDM, the parameter $\alpha$ of the Mittag-Leffler function has a degree of freedom $(0< \alpha < 1)$. Therefore, the following discussion of the change in the $^{137}$Cs activity concentration of the lake water over time will be compared between the FDM and the TDM.

**Figure 2:**
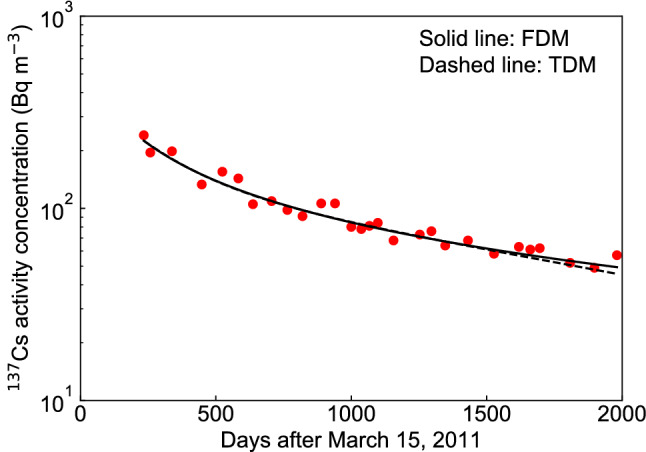
Comparison of blind test between the fractional diffusion model (FDM) and the two-component decay function model (TDM) using the measured data (234–1527 days after March 15, 2011) for the $^{137}$Cs activity concentrations of water in Lake Onuma. The solid red circles indicate the measured $^{137}$Cs activity concentration found in the supplementary data^10^.

We fitted the FDM and the TDM to the measured values 234–1527 days after March 15, 2011, predicted the ^137^Cs activity concentration at 1527–1981 days, and then compared the predicted values and the measured values between the FDM and the TDM (see Fig. 2). The values of $\epsilon ^{2}$ for the FDM and the TDM were 0.215 and 0.251, respectively and slightly better agreement was found for the FDM than for the TDM.

The fitting formula based on the TDM^10^ consisted of the sum of two Mittag-Leffler functions with $\alpha$ = 1. Noting that $E_{1} (\pm {z}) = \exp (\pm {z})$, Eq. (40) can be written using the two Mittag-Leffler functions as follows:

49 $$\begin{aligned} C_{TDM} (t) = Q_{1} \, E_{1} (- k_{1} t ) + Q_{2} \, E_{1} (- k_{2} t ). \end{aligned}$$

The FDM can reproduce the long-tailed distribution with respect to time *t* with a single Mittag-Leffler function. The TDM loses the degrees of freedom of $\alpha$ because the value of $\alpha$ is fixed to one. Therefore, at least two Mittag-Leffler functions must be used to reproduce the apparent long-tailed distribution.

**Figure 3:**
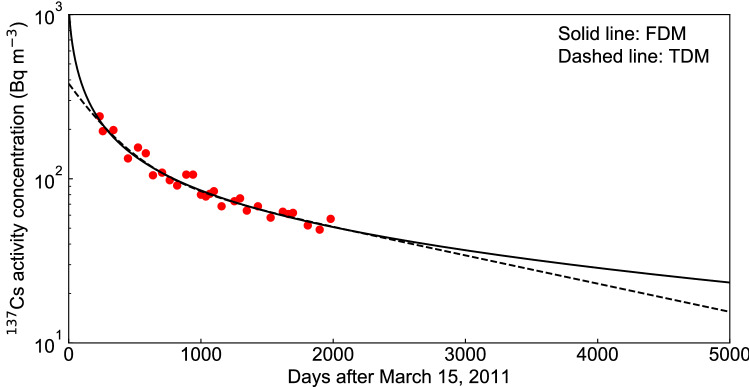
Comparison between the fractional diffusion model (FDM) and the two-component decay function model (TDM) for changes in the $^{137}$Cs activity concentrations of water in Lake Onuma over 5000 days from March 15, 2011. The solid red circles indicate the measured $^{137}$Cs activity concentration found in the supplementary data^10^.

A comparison of the FDM and the TDM predictions for $^{137}$Cs activity concentrations in the lake water up to 5000 days after the accident (see Fig. 3) suggested that the FDM prediction indicates a prolonged low-level radioactive contamination after 2000 days compared to the TDM prediction. The squared relative errors of the FDM and the TDM were 0.207 and 0.208, respectively, and there were no large differences in the prediction results up to 3000 days after the accident. The initial radioactivity of the lake water was 380 Bq ${\text {m}}^{-3}$ in the TDM prediction, while it was 1256 Bq ${\text {m}}^{-3}$ in the FDM prediction. Most of the $^{137}$Cs in Lake Onuma was remained within the uppermost layer (5 cm) of the deposits on the lake bottom. The inventory of $^{137}$Cs in the sediment has been estimated at 20 kBq per unit area (${\text {m}}^{2}$) of the lake bottom^49,50^. We multiply the value, 20 kBq ${\text {m}}^{-2}$, by the bottom area of the lake^51^, then divide it by the total volume of the lake water^51^. Thereby we obtain the estimation of the initial activity concentration 1960 Bq ${\text {m}}^{-3}$. Comparing initial value of our data (1960 Bq ${\text {m}}^{-3}$) with the result of the FDM (*A* = 1256 Bq ${\text {m}}^{-3}$), the predictions of the FDM at $t = 0$ was in relatively good agreement. On the other hand, after 2000 days, the TDM prediction showed an exponential decrease in $^{137}$Cs activity concentration, while the FDM prediction showed a gradual decrease in $^{137}$Cs activity concentration. The difference between the TDM and the FDM is due to the dominance of the second term ($Q_{2} \exp (- k_{2} t )$) in Eq. (40) after 2000 days. On the other hand, in the FDM prediction Eq. (48) holds when the time *t* becomes large and the $^{137}$Cs activity concentration follows the power law of *t*, so that the decrease of $^{137}$Cs becomes gradual.

**Figure 4:**
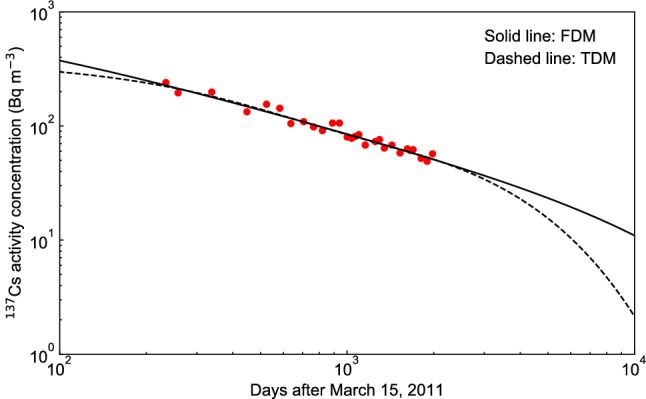
Double logarithmic plot for comparison between the fractional diffusion model (FDM) and the two-component decay function model (TDM) for changes in the $^{137}$Cs activity concentrations of water in Lake Onuma over 10,000 days from March 15, 2011. The solid red circles indicate the measured $^{137}$Cs activity concentration found in the supplementary data^10^.

**Figure 5:**
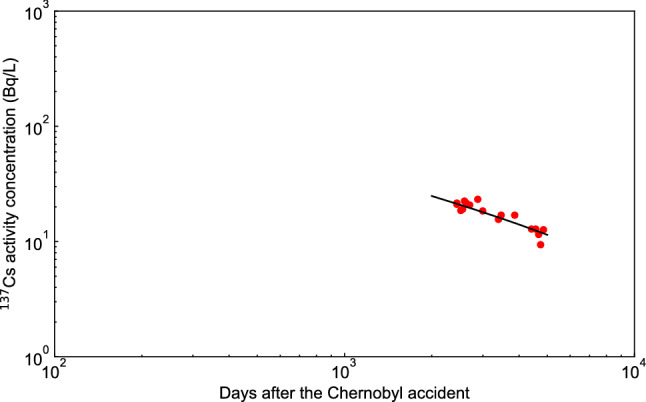
Double logarithmic plot for comparison between the measured values by Bulgakov et al.^11^ and the fractional diffusion model (FDM) for changes in the $^{137}$Cs activity concentrations of water in Svyatoe lake in the Bryansk region of Russia days after the Chernobyl accident 2400–4900 days (7–13 years) . The solid line indicates the FDM using $\alpha = 0.62$, $\xi = 5.00$, $\tau = 693$ in Eq. (39) and red circles indicate the measured $^{137}$Cs activity concentration shown in Fig. 3 in Bulgakov et al.^11^.

To clarify the characteristics between the FDM and the TDM, we plotted the double logarithmic plot of both prediction results up to 10,000 days after the accident (see Fig. 4), and the FDM prediction suggested that the low-level $^{137}$Cs contamination was prolonged than the TDM prediction. The TDM prediction shows a rapid decrease in $^{137}$Cs activity concentration after 4000 days after the accident, while the FDM prediction values decreased linearly in double logarithmic plot until 10,000 days. Hence the prediction of the FDM decreased in inverse proportion to the power of time ($t^{- \alpha }$), suggesting that the contamination of low-level $^{137}$Cs is prolonged. Although the measured data for Lake Onuma is only available until 1981 days after March 15, 2011, the comparison of the FDM with the measured data for Svyatoe lake in the Bryansk region of Russia^11^, which is a closed lake, suggested that the FDM could be applied to the prediction of Lake Onuma 2000–5000 days after the Fukushima accident. Bulgakov et al.^11^ compared the measured $^{137}$Cs activity concentrations of the water in Svyatoe lake during the period of about 2400–4900 days (about 7–13 years) after the Chernobyl accident with the results of calculations using Eq. (42) and showed a good agreement. Therefore, in order to verify the validity of the prediction of $^{137}$Cs activity concentration in Lake Onuma after 2000–5000 days after March 15, 2011 using the FDM, the fitting parameters obtained from Lake Onuma ($\alpha = 0.62$, $\xi = 5.00$, $\tau = 693$) were substituted into Eq. (39) and the fitting parameter *A* was fitted to the measured data of the water in Svyatoe lake^11^ from 2400 to 4900 days and compared with the measured value (see Fig. 5). Both the predicted values based on the FDM and the values measured by Bulgakov et al.^11^ decreased with the power law of time, and the two values agreed well. Therefore, the prediction of the FDM is considered to be valid for this period in Lake Onuma. The validity of the prediction for 5000–10,000 days after the accident needs to be evaluated by future measurements.

**Figure 6:**
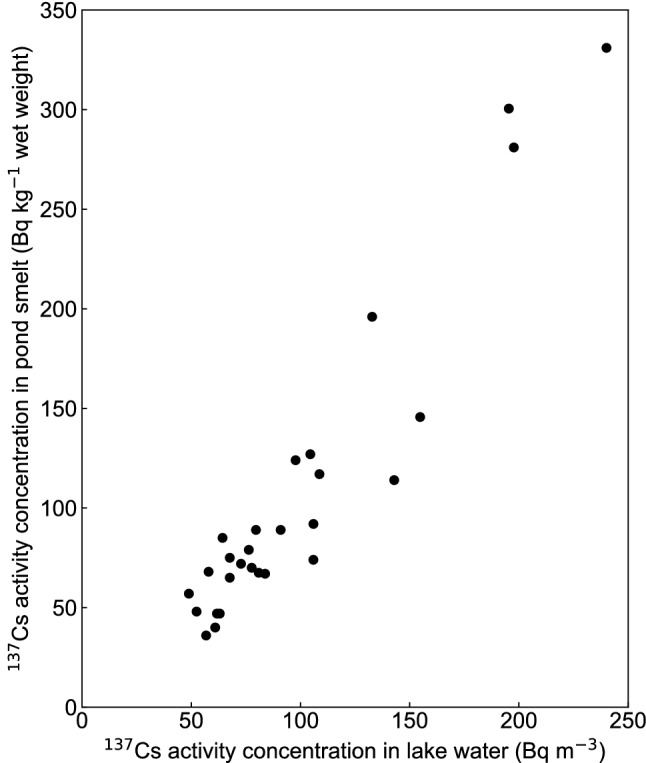
Correlations of the $^{137}$Cs activity concentrations between lake water and pond smelt in Lake Onuma. The calculated value of correlation coefficient was 0.95.

On the other hand, the correlation coefficient between the $^{137}$Cs activity concentration (Bq ${\text {m}}^{-3}$) of the water in Lake Onuma and the $^{137}$Cs activity concentration (Bq ${\text {kg}}^{-1}$ wet weight) of pond smelt (Hypomesus nipponensis), a typical fish species inhabiting the lake, was 0.95, indicating a strong positive correlation (Fig. 6). The activity concentration of $^{137}$Cs of pond smelt decreased rapidly from August 2011 (160 days after March 15, 2011) to September 2012 (552 days), but showed a gradual decreasing trend after October 2012 (592 days)^10^. This trend is consistent with the change in the $^{137}$Cs activity concentration of the lake water in Lake Onuma. If the correlation coefficient between the $^{137}$Cs activity concentration of the lake water and the $^{137}$Cs activity concentration of pond smelt is close to one, the $^{137}$Cs activity concentration of pond smelt can be predicted by applying the FDM. Therefore, the correlation coefficient between the $^{137}$Cs activity concentration of the lake water and the $^{137}$Cs activity concentration of pond smelt was calculated, and a strong correlation (correlation coefficient = 0.95) was found.

**Figure 7:**
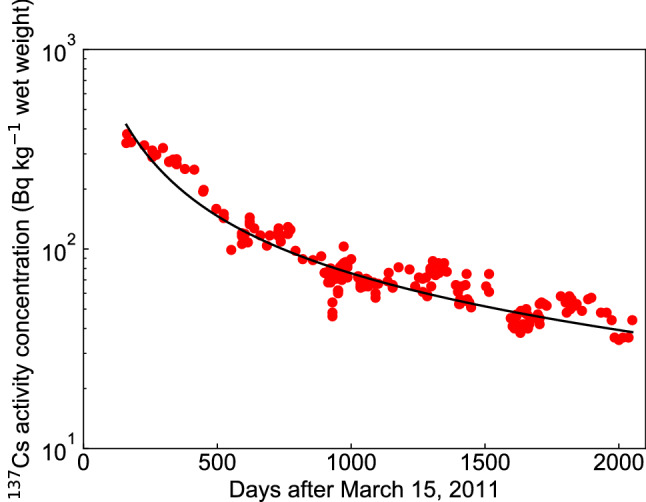
Change in the $^{137}$Cs activity concentration of pond smelt in Lake Onuma using the fractional diffusion model (FDM). The solid red circles indicate the measured $^{137}$Cs activity concentration in pond smelt found in the supplementary data^10^.

We compared the fitting results of $^{137}$Cs activity concentrations of pond smelt in the lake water using the FDM and the TDM, and found that both the FDM fitting (Fig. 7, $\epsilon ^{2} = 5.60$) and the TDM fitting ($\epsilon ^{2} =4.96$) agreed with the measured values. In the following discussion, we compare the predictions of the FDM and the TDM.

**Figure 8:**
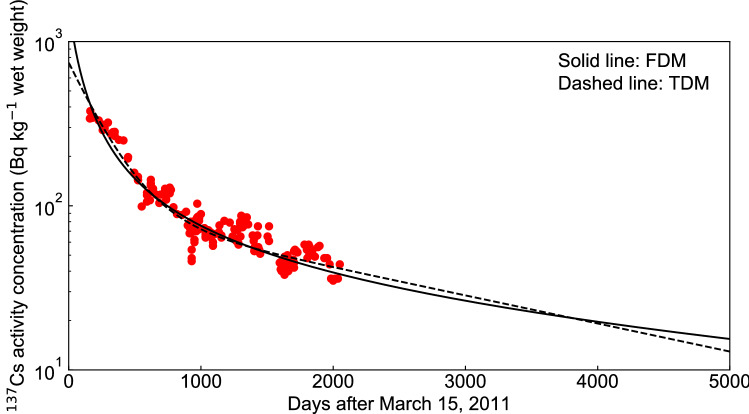
Comparison between the fractional diffusion model (FDM) and the two-component decay function model (TDM) for changes in the $^{137}$Cs activity concentrations of pond smelt in Lake Onuma over 5000 days from March 15, 2011. The solid red circles indicate the measured $^{137}$Cs activity concentration found in the supplementary data^10^.

As shown in Fig. 8, there was no clear difference between the FDM and the TDM in the prediction of $^{137}$Cs activity concentration of pond smelt from 2000 to 4000 days, but the FDM prediction showed a gradual decrease after 4000 days. The reason for this difference is that the TDM decreases exponentially, while the FDM decays with the power law of time.

**Figure 9:**
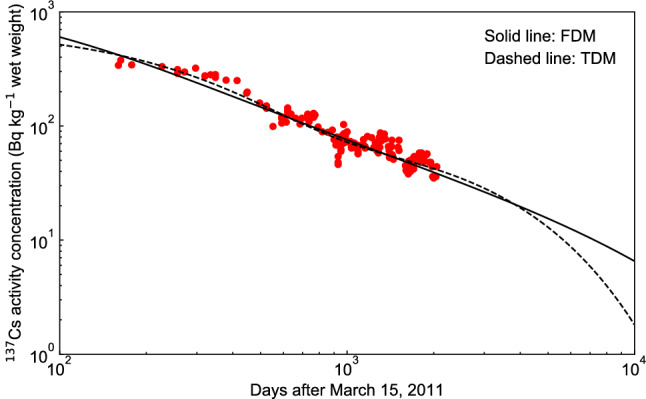
Double logarithmic plot for comparison between the fractional diffusion model (FDM) and the two-component decay function model (TDM) for changes in the $^{137}$Cs activity concentrations of pond smelt in Lake Onuma over 10,000 days from March 15, 2011. The solid red circles indicate the measured $^{137}$Cs activity concentration found in the supplementary data^10^.

As shown in Fig. 9, double logarithmic plot for the predictions of $^{137}$Cs activity concentration of pond smelt up to 10,000 days showed that the FDM prediction resulted in a longer period of low-level $^{137}$Cs contamination than the TDM prediction. In the FDM prediction, when time *t* $\gg$ $\tau$, the $^{137}$Cs activity concentration decreased according to the power law $t^{- \alpha }$, whose index was $\alpha =0.76$. This indicated that decrease of the low-level $^{137}$Cs activity concentration of pond smelt was prolonged because the $^{137}$Cs activity concentration decreased gradually according to the power law of time. In other words, the results suggested that the low-level $^{137}$Cs contamination of pond smelt was also persistent due to the influence of $^{137}$Cs activity concentration in the lake water.

## Conclusion

In conclusion, we showed that using the FDM for long-term prediction of $^{137}$Cs activity concentration in Lake Onuma, a closed lake, prolongs the low-level radioactive contamination compared to the TDM, a conventional fitting model. We derived the Mittag-Leffler function as a solution of the fractional diffusion equation based on the FDM in order to reproduce the gradual decrease of $^{137}$Cs activity concentration in closed lakes. This function has the property of exponential function when time *t* is small, and becomes long-tailed distribution when time *t* becomes large because it follows the power law $t^{- \alpha }$. Using the FDM based formula (Mittag-Leffler function), it was possible to fit the temporal variation in the measured $^{137}$Cs activity concentration in the lake water without using formula consisting of two component exponential functions. The long-term prediction 5000 days after the accident using the fitting parameters to the measured data suggested that the FDM would result in a prolonged contamination with low activity concentrations of $^{137}$Cs compared to the TDM. Although the measured data for Lake Onuma is available until 1981 days after the accident, the comparison of the FDM calculation with the measured data for Svyatoe lake in Russia (about 2400–4900 days after the Chernobyl accident), which is a closed lake, suggested that the FDM could be applied to the prediction of Lake Onuma 2000–5000 days after the Fukushima accident. The validity of the prediction for 5000–10,000 days after the Fukushima accident needs to be evaluated by measurement in the future. The correlation coefficient between the $^{137}$Cs activity concentration of the lake water and the $^{137}$Cs activity concentration of pond smelt was calculated, and a strong positive correlation (correlation coefficient = 0.95) was found. Because the fitting with the measured $^{137}$Cs activity concentration of pond smelt showed a good agreement, we performed long-term prediction of $^{137}$Cs activity concentration in pond smelt using the FDM. A comparison of the FDM prediction with the TDM prediction suggested that the FDM prediction showed prolonged low-level $^{137}$Cs contamination. The prediction can be improved by applying the sum of three or more exponential functions instead of the sum of two exponential functions as in the TDM. Since no monitoring has been carried out since 2000 days after the accident, it makes sense to use the prediction formulae to give the residents living in the vicinity in Lake Onuma an idea of the future prospects of radioactive contamination. On the other hand, the monitoring of radiocesium is a research topic that should be continued in the future. To be summarized, a combination of the exponential functions to describe $^{137}$Cs dynamics in a lake for initial/short-term phase and the power law (diffusional) approach for mid-, and long-term is the merit of the proposed fitting model. The disadvantage of the FDM is that the fitting parameters of the FDM are less related to physicochemical processes in a lake and their characteristics than the models of Smith et al.^13^ and Konoplev et al.^18,19^.
